# Streptozotocin-Induced Early Thermal Hyperalgesia is independent of Glycemic State of Rats: Role of Transient Receptor Potential Vanilloid 1(TRPV1) and Inflammatory mediators

**DOI:** 10.1186/1744-8069-7-52

**Published:** 2011-07-27

**Authors:** Mahendra Bishnoi, Christine A Bosgraaf, Mruvil Abooj, Linlin Zhong, Louis S Premkumar

**Affiliations:** 1 Department of Pharmacology, Southern Illinois University School of Medicine, Springfield, IL-62702, USA

**Keywords:** Inflammation, microglia, resiniferatoxin, streptozotocin, TRPV1

## Abstract

**Background:**

Streptozotocin (STZ) is used as a common tool to induce diabetes and to study diabetes-induced complications including diabetic peripheral neuropathy (DPN). Previously, we have reported that STZ induces a direct effect on neurons through expression and function of the Transient receptor potential vanilloid 1 (TRPV1) channel in sensory neurons resulting in thermal hyperalgesia, even in non-diabetic STZ-treated mice. In the present study, we investigated the role of expression and function of TRPV1 in the central sensory nerve terminals in the spinal cord in STZ-induced hyperalgesia in rats.

**Results:**

We found that a proportion of STZ-treated rats were normoglycemic but still exhibited thermal hyperalgesia and mechanical allodynia. Immunohistochemical data show that STZ treatment, irrespective of glycemic state of the animal, caused microglial activation and increased expression of TRPV1 in spinal dorsal horn. Further, there was a significant increase in the levels of pro-inflammatory mediators (IL-1β, IL-6 and TNF-α) in spinal cord tissue, irrespective of the glycemic state. Capsaicin-stimulated release of calcitonin gene related peptide (CGRP) was significantly higher in the spinal cord of STZ-treated animals. Intrathecal administration of resiniferatoxin (RTX), a potent TRPV1 agonist, significantly attenuated STZ-induced thermal hyperalgesia, but not mechanical allodynia. RTX treatment also prevented the increase in TRPV1-mediated neuropeptide release in the spinal cord tissue.

**Conclusions:**

From these results, it is concluded that TRPV1 is an integral component of initiating and maintaining inflammatory thermal hyperalgesia, which can be alleviated by intrathecal administration of RTX. Further, the results suggest that enhanced expression and inflammation-induced sensitization of TRPV1 at the spinal cord may play a role in central sensitization in STZ-induced neuropathy.

## Background

STZ, a glucosamine-nitrosourea compound obtained from *Streptomyces achromogenes*, is used as an experimental tool to develop animal models to study diabetes and associated complications that include diabetic peripheral neuropathy (DPN). Several mechanisms, including increase in oxidative/nitrosative stress have been proposed for STZ-induced β-cell death. Further, the ability of STZ to act as a NO donor has led many investigators to postulate that NO is involved in STZ-induced β-cell death [1-3]. Recently, it has been proposed that pancreatic β-cells are selectively sensitive to STZ, because they contain higher levels of *O*-GlcNAc-selective *N*-acetyl-b-d-glucosaminidase (OGIcNAcase, an enzyme responsible for attaching *O*GlcNAc to proteins) than any other cell type [4-6]. STZ has been shown to inhibit the enzyme OGlcNAcase, which causes cytotoxicity selectively to β-cells [4,6].

DPN has been characterized by early thermal and mechanical hyperalgesia in different animals models [7,8]. It has been proposed that STZ-induced hyperglycemia contribute to the development of hyperalgesia [7,9], but it is becoming evident that factors other than hyperglycemia may be involved in development of early hyperalgesia following STZ treatment [10-13]. Studies have suggested that intracerebroventricular administration of STZ can cause behavioral alterations and changes in brain pathology independent of changes in β-cells [14]. Previous studies from our laboratory have shown that STZ exerts a direct action on dorsal root ganglion (DRG) neurons altering the expression and function of TRPV1 via the reactive oxygen species (ROS)-p38 Mitogen Activated Protein Kinase (MAPK) pathway in mice [12]. In *in-vitro *studies, STZ-treated neurons exhibited an increase in TRPV1-mediated currents through the ROS-mediated pathway. We further observed that STZ also caused an increase in the phosphorylated form of p38 MAPK, suggesting that the increase in the TRPV1 protein expression may involve the ROS-p38 MAPK pathway [12]. All these changes were independent of glycemic state of the animals.

There is growing evidence that supports a prominent role of inflammation in the development and maintenance of neuropathic pain. Microglia and astrocyte activation is observed in the spinal cord following PNS/CNS injury and STZ-induced neuropathic pain [15-18]. Analgesic effect of inhibitors of phosphorylation of the MAPKs and Extracellular Signal-regulated Protein Kinase (ERK) [18-20] further substantiate the role of inflammation in STZ-induced neuropathic pain. Activated microglia and their interaction with neurons in spinal cord are associated with the development and maintenance of neuropathic pain [21,22]. Enhanced levels of pro-inflammatory cytokines and neuroinflammation can sensitize TRPV1 and increase the pain sensitivity [23,24]. On the other hand, stimulation of TRPV1 can mediate the release of pro-inflammatory cytokines, neuropeptides (SP and CGRP), and glutamate through influx of Ca^2+ ^in the activated microglia. These changes may underlie the development of central sensitization leading to neuropathic pain [25].

Resiniferatoxin (RTX) is a potent TRPV1 agonist and its analgesic actions can be explained by its ability to cause depolarization block of the peripheral or central terminals in the short-term and nerve terminal ablation in the long-term [26-29]. Earlier studies have shown that intrathecal and intraganglionic administration of RTX induced long-lasting analgesia suggesting the possibility of nerve terminal ablation [27,29-31]. In dorsal root ganglion and dorsal horn (DRG-DH) neuronal co-cultures and in acute spinal cord or caudal spinal trigeminal nucleus slice preparations, RTX enhanced frequency of spontaneous and miniature excitatory post synaptic currents (mEPSCs), without affecting their amplitude, suggesting a presynaptic locus of action [27,32]. However, evoked synaptic currents were inhibited due to slow and sustained activation of presynaptic TRPV1 leading to depolarization block by maintaining the Na^+ ^channels in an inactivated state at the central sensory nerve terminals [27].

This study was undertaken to investigate the role of TRPV1 expressed in the central sensory nerve terminals of the spinal cord following intraperitoneal administration of STZ. We have found that STZ-induced thermal and mechanical hyperalgesia were independent of glycemic state of the animal. STZ exerted a direct effect by inducing enhanced expression of TRPV1 and a resultant increase in TRPV1-mediated neuropeptide release. STZ also caused microglial activation in the spinal cord and increased the levels of inflammatory mediators. Intrathecal administration of RTX decreased TRPV1-mediated neuropeptide release and alleviated thermal hypersensitivity without affecting mechanical hypersensitivity.

## Results

### Role of intraperitoneal administration of STZ on various metabolic parameters in rats

**Figure 1:**
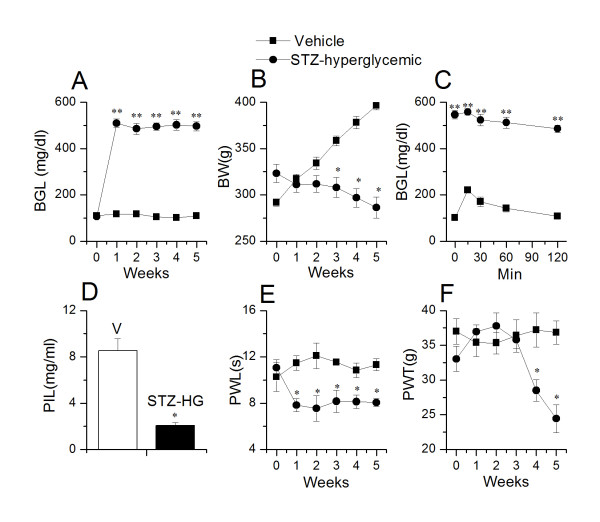
**Changes in blood glucose levels, body weight, glucose tolerance, plasma insulin level, thermal pain sensitivity, mechanical allodynia in STZ-treated hyperglycemic (STZ-HG) rats**. **A**. Within a week after intraperitoneal injection of STZ, blood glucose levels (BGL) significantly increased (p < 0.001) and remained constant throughout the course of the experiment as compared to vehicle-treated rats. **B**. The body weight of STZ-HG rats (filled circles, n = 18) decreased, whereas body weight of vehicle-treated rats (filled squares, n = 10) increased steadily over the course of 5 weeks. **C**. Glucose tolerance test (GTT) in STZ-HG and vehicle-treated rats 5 weeks after STZ administration. **D**. Plasma insulin levels were significantly lower (p < 0.05) in STZ-HG rats as compared to vehicle-treated rats. **E**. STZ-HG rats exhibited a significant decrease in PWL (p < 0.05) as compared to vehicle-treated rats. **F**. STZ-HG rats exhibited a significant (p < 0.05) decrease in PWT after 3 weeks of STZ administration as compared to vehicle-treated rats. Asterisks (*, **) represent p < 0.05 and p < 0.001, respectively.

**Figure 2:**
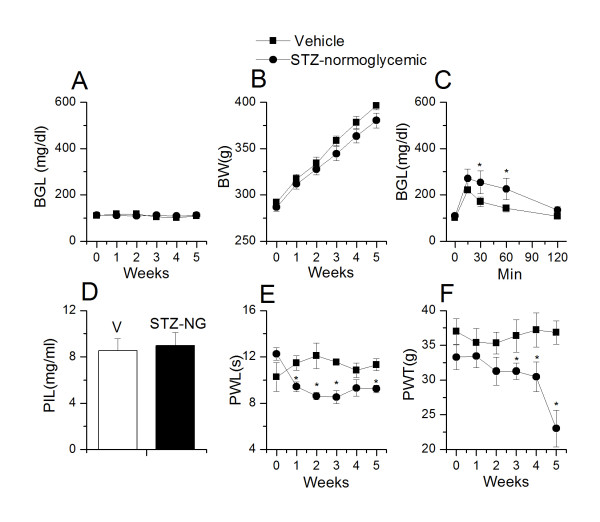
**Changes in blood glucose levels, body weight, glucose tolerance, plasma insulin levels, thermal pain sensitivity, mechanical allodynia in STZ-treated normoglycemic (STZ-NG) rats**. **A**. Blood glucose levels (BGL) remained constant throughout the course of the experiment in STZ-NG and vehicle-treated rats. **B**. The body weight of STZ-NG rats (filled circles, n = 12) and vehicle-treated rats (filled squares, n = 10) increased steadily over the course of 5 weeks **C**. Glucose tolerance test (GTT) in STZ-NG and vehicle-treated rats after 5 weeks of STZ administration. **D**. Plasma insulin levels were unchanged in STZ-NG rats as compared to vehicle-treated rats. **E**. STZ-NG rats exhibited a significant decrease (p < 0.05) in PWL as compared to vehicle-treated rats. **F**. STZ-NG rats exhibited a significant (p < 0.05) decrease in PWT after 3 weeks of STZ administration as compared to vehicle-treated rats. Asterisk (*) represent p < 0.05.

STZ is commonly used to induce diabetes in rodents to study diabetes and related complications including DPN. In this study, STZ (55 mg/kg, i.p.) was administered to rats to induce diabetes. After 24 hour of STZ treatment, 60% animals were hyperglycemic (STZ-HG, n = 24/40) (blood glucose levels > 300 mg/dl (range = 426-534 mg/dl), whereas, rest of the animals were normoglycemic (STZ-NG, n = 16/40) (blood glucose levels < 120 mg/dl (range = 95-118 mg/dl). There was no change in the glycemic state of animals over a period of 5 weeks (Week 5: vehicle treated, 109.3 ± 3.13 (n = 5); STZ-HG, 496.4 ± 19.87 (n = 12); STZ-NG, 111.5 ± 2.98 mg/dl (n = 8) (Figure 1A, 2A). Increase in body weight was inversely correlated with the increase in blood glucose levels. STZ-HG animals did not show a significant change in the body weight over the 5 week period (Week 1, 323.2 ± 9.7 vs. week 5, 286.5 ± 11.2 g, n = 24), whereas STZ-NG animals showed significant increase in body weight over the 5 week period (Week 1, 282.2 ± 4.3 vs. week 5, 380.3 ± 8.0 g, n = 16, p < 0.01), similar to that seen in vehicle-treated animals (Week 1, 291.7 ± 4.0 vs. week 5, 396.4 ± 4.5 g, n = 10, p < 0.01) (Figure 1B, 2B). In order to further confirm the glycemic status, we performed glucose tolerance test (GTT) and measured plasma insulin levels in vehicle-treated, STZ-HG and STZ-NG animals after 5 weeks. In a GTT, administration of glucose (1 g/kg i.p.) significantly increased the blood glucose levels (101.2 ± 3.1 vs. 220.0 ± 2.6 mg/dl, n = 6, p < 0.02) in vehicle-treated animals, which became normal after 2 h (107.2 ± 3.1 mg/dl). Blood glucose levels remained significantly higher in STZ-HG group (> 500 mg/dl, n = 6, at all time points (0-2 h) (Figure 1C). In STZ-NG group, i.p. administration of glucose (1 g/kg) significantly increased blood glucose levels (101.2 ± 3.1 vs. 270.0 ± 40.9 mg/dl, n = 6, p < 0.01), and remained higher than the vehicle-treated animals (Figure 2C) suggesting a slightly compromised β-cell function. Plasma insulin levels were significantly lower in STZ-HG animals as compared to vehicle-treated animals (vehicle treated, 8.5 ± 1.0, STZ-HG, 2.0 ± 0.2 mg/ml, n = 6, p < 0.05) (Figure 1D). There was no significant difference in insulin levels between STZ-NG and vehicle-treated animals (vehicle-treated, 8.5 ± 1.0, STZ-NG, 8.9 ± 1.1 mg/ml, n = 6, ns) (Figure 2D). From these results, we conclude that STZ causes a substantial damage to pancreatic β-cells indicated by the blood glucose levels and GTT, whereas in a significant number of animals the β-cell damage may not have been as severe indicated by the blood glucose levels following GTT.

### Intraperitoneal administration of STZ and early thermal hyperalgesia and mechanical allodynia in rats

Next, in order to determine the effect of glycemic/metabolic state on thermal hyperalgesia and mechanical allodynia, we divided the animals in 3 different groups (vehicle-treated, STZ-HG and STZ-NG). Interestingly, we found that both STZ-HG and STZ-NG groups exhibited significant increase in thermal hyperalgesia as compared to vehicle-treated animals after 1 week of STZ treatment (vehicle, week 0: 10.2 ± 1.2 s;week 1: 11.8 ± 0.6 s, n = 6 (p > 0.05); STZ-HG, week 0: 11.0 ± 0.6 s; week 1: 7.8 ± 0.5 s, n = 12 (p < 0.05); STZ-NG, week 0: 12.2 ± 0.5 s; week 1: 9.4 ± 0.4 s, n = 8 (p < 0.05) (Figure 1E, 2E). Animals were hyperalgesic over the duration (5 weeks) of the study (vehicle, 11.3 ± 0.5 s; STZ-HG, 8.0 ± 0.3 s; STZ-NG, 9.2 ± 0.3 s (p < 0.05); although towards the end there was a trend that hyperalgesic state was reversing to become normal. Reports from other laboratories and ours have suggested that STZ causes an early phase of thermal hyperalgesia (upto 5-6 week) followed by a late phase of hypoalgesia (after 6 week) [12,33,34]. Mechanical allodynia developed after 3 weeks and the trend was similar in STZ-HG and STZ-NG animals. After 5 weeks of STZ treatment, animals showed significant increase in mechanical allodynia as compared to vehicle-treated animals (vehicle, week 0: 37.0 ± 1.8 g; week 5: 36.8 ± 1.6 g, n = 6(p > 0.05); STZ-HG, week 0: 33.0 ± 1.8 g; week 5: 24.4 ± 2.0 g, n = 12 (p < 0.02); STZ-NG, week 0: 33.2 ± 1.7 g; week 5: 23.0 ± 2.6 g, n = 8 (p < 0.02) (Figure 1F, 2F). It is generally believed that thermal hyperalgesia and mechanical allodynia are due to hyperglycemia. From our studies, it is clear that early phase of thermal and mechanical hyperalgesia is independent of glycemic state.

### Intraperitoneal administration of STZ and microglial activation and TRPV1 expression in spinal dorsal horn

**Figure 3:**
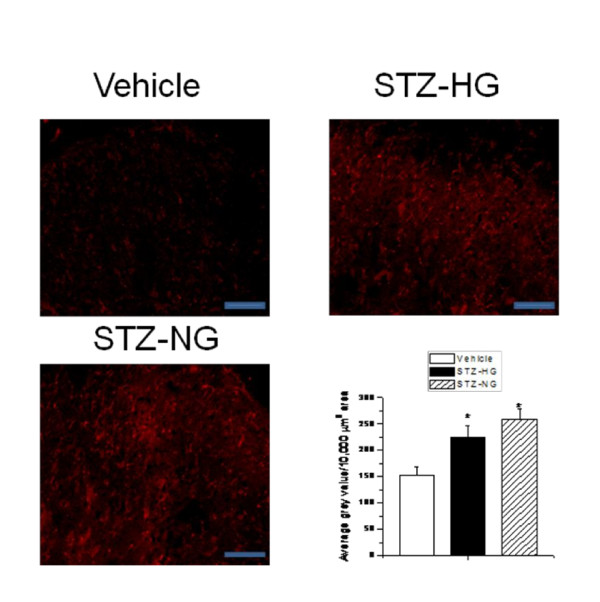
**Altered microglial activation in spinal cord dorsal horn of STZ-treated rats**. **A**. Representative images of OX-42 staining (marker for microglial activation) from a vehicle-treated, STZ-HG and STZ-NG rats. **B**. Average gray values/10,000 μm^2 ^area of OX-42 staining in spinal dorsal horn was significantly increased (p < 0.05) in both STZ-HG and STZ-NG rats as compared to vehicle-treated rats. Asterisk (*) represents p < 0.05. Scale bar is 50 μm.

**Figure 4:**
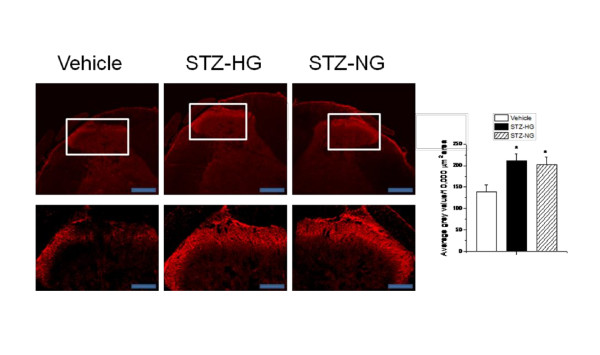
**Altered TRPV1 staining in spinal cord dorsal horn of STZ-treated rats**. **A**. Representative images of TRPV1 staining from a vehicle-treated, STZ-HG and STZ-NG rats. An enlarged segment has also been shown. **B**. Average gray values/10,000 μm^2 ^area of TRPV1 staining in dorsal horn was significantly increased (p < 0.05) in both STZ-HG and STZ-NG rats as compared to vehicle-treated rats. Asterisk (*) represents p < 0.05. Scale bar is 200 μm and 50 μm for upper and lower panels, respectively.

In order to correlate the development of early phase of thermal and mechanical hyperalgesia with the changes in the spinal cord, we performed immunohistochemistry to determine microglial activation (staining for OX-42) and relative TRPV1 expression in spinal dorsal horn tissue in vehicle-treated, STZ-HG and STZ-NG animals. There are reports suggesting the involvement of inflammation, microglial activation and increased TRPV1 expression and function in STZ-induced hyperalgesia [12,16,18]. Following i.p. administration of STZ, the intensity of OX-42 staining increased significantly in the laminae I and II of spinal dorsal horn in both STZ-HG and STZ-NG animals as compared to vehicle-treated animals (Figure 3). STZ treatment also significantly increased the TRPV1 staining in central terminals of spinal dorsal horn as compared to vehicle-treated animals (Figure 4). The intensity of TRPV1 activation was similar in both STZ-HG and STZ-NG animals. STZ-induced microglial activation and increased TRPV1 expression in spinal dorsal horn is independent of glycemic state of animals. These results suggest that the effects are possibly due to direct actions of STZ rather than as a result of hyperglycemia.

### Intraperitoneal administration of STZ and spinal pro-inflammatory cytokines

**Figure 5:**
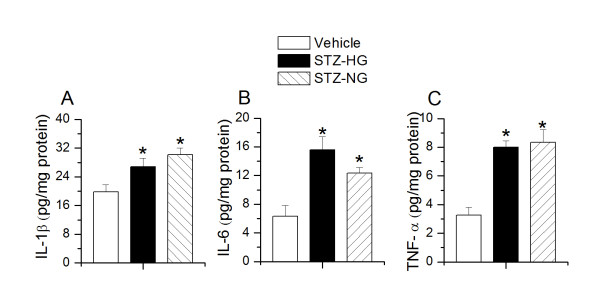
**Effect of intraperitoneal administration of STZ on spinal pro-inflammatory cytokine levels**. Interleukin-1β(IL-1β)**(A)**, Interleukin-6(IL-6)**(B)**, Tumor necrosis factor-α (TNF-α)**(C) **in lumbar spinal cord tissue homogenates. Asterisk (*) represent p < 0.05 as compared to vehicle-treated rats.

Next, we determined the role of pro-inflammatory mediators in manifesting these responses. I.p. administration of STZ significantly increased the levels of pro-inflammatory cytokines (IL-1β, IL-6, TNF-α) in the spinal cord homogenates of rats, which were not related to the glycemic state of the animals (IL-1β: vehicle,19.7 ± 1.9; STZ-HG, 26.8 ± 2.4; STZ-NG, 30.1 ± 1.8 pg/mg protein, n = 5, p < 0.05, Figure 5A; IL-6: vehicle, 6.3 ± 1.5; STZ-HG,15.6 ± 1.8; STZ-NG,12.3 ± 0.8 pg/mg protein, n = 5, p < 0.05, Figure 5B; TNF-α: vehicle, 3.2 ± 0.5; STZ-HG, 7.9 ± 0.4; STZ-NG, 8.3 ± 0.8 pg/mg protein, n = 5, p < 0.05, Figure 5C. We suggest that the increases in pro-inflammatory mediators are due to microglial activation or increased TRPV1 expression in spinal dorsal horn, but not due to STZ-induced hyperglycemic state.

### TRPV1-mediated CGRP release and intraperitoneal administration of STZ

**Figure 6:**
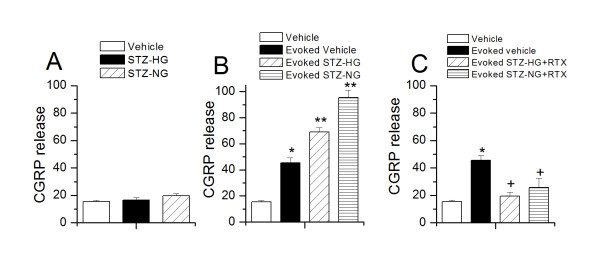
**Change in basal and capsaicin-evoked CGRP release in STZ-treated rats A. Basal CGRP release in spinal dorsal horn was unaffected after STZ treatment B**. Both STZ-HG and STZ-NG rats show a significant increase in the capsaicin evoked-CGRP release **C**. Intrathecal RTX treatment prevent the increase in capsaicin evoked-CGRP release in STZ-treated rats. Asterisks (*, ^+^) represent p < 0.05 as compared to vehicle and evoked vehicle-treated rats respectively.

As indicated earlier that both peripheral and central terminals of the sensory neurons express TRPV1. Following the observation that STZ treatment increased the intensity of TRPV1 staining in spinal dorsal horn, we hypothesized that STZ administration might affect TRPV1-mediated functions such as neuropeptide release in central terminals of DRG neurons. TRPV1-mediated CGRP release is a reliable assay to determine TRPV1 functions. The neuropeptide release was normalized to the weight of the spinal cord tissue. STZ treatment did not cause a significant change in basal CGRP release in lumbar spinal region (vehicle,15.5 ± 0.9; STZ-HG,16.0 ± 2.1; STZ-NG, 19.65 ± 1.5 ng/g tissue/5 min, n = 5 (p > 0.05) (Figure 6A). However, STZ treatment significantly increased capsaicin (10 μM)-evoked CGRP release in the lumbar spinal cord tissue (basal vehicle, 15.5 ± 0.9, evoked vehicle, 45.4 ± 3.6 (p < 0.05 as compared to basal vehicle); evoked STZ-HG, 69.1 ± 3.2 (p < 0.05 as compared to evoked vehicle); evoked STZ-NG, 95.3 ± 5.9 (p < 0.05 as compared to evoked vehicle) ng/g tissue/5 min, (n = 5) (Figure 6B). The changes in basal and capsaicin-evoked CGRP release were independent of glycemic state of the animals and that these changes are positively correlated with the expression of TRPV1 in the spinal dorsal horn. In order to confirm the involvement of TRPV1, we determined CGRP release following intrathecal administration of RTX, which is known to specifically target TRPV1 and abolish the effects mediated by TRPV1. Intrathecal administration of RTX prevented the increase in the capsaicin-evoked CGRP release in STZ-treated animals (basal vehicle, 15.5 ± 0.9, evoked vehicle, 45.4 ± 3.6 (p < 0.05 as compared to basal vehicle); evoked STZ-HG (RTX-treated), 19.4 ± 2.8 (p > 0.05 as compared to evoked vehicle); evoked STZ-NG, 25.6 ± 6.8 (p > 0.05 as compared to evoked vehicle) ng/g tissue/5 min, (n = 5) (Figure 6C).

### Role of intrathecal RTX on STZ-induced increase in thermal and mechanical hyperalgesia

**Figure 7:**
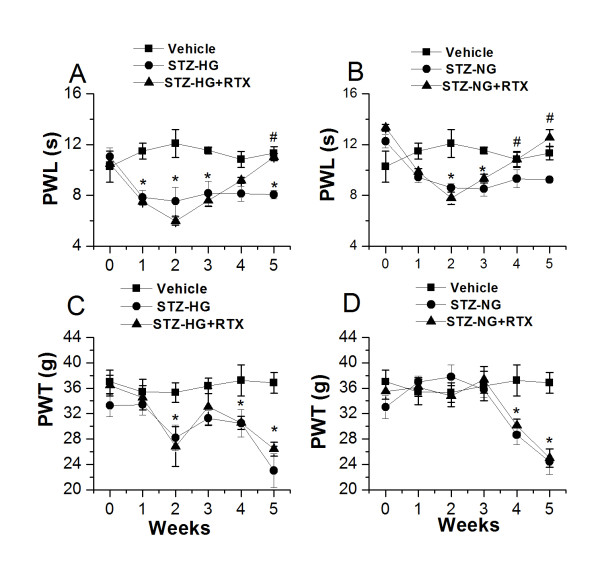
**Effect of intrathecal administration of RTX on STZ-induced thermal hyperalgesia and mechanical allodynia**. **A.B**. RTX attenuated thermal hyperalgesia in both STZ-HG and STZ-NG rats. **C.D**. RTX administration 2 weeks after STZ injection did not have any effect on mechanical allodynia in both STZ-HG and STZ-NG rats. Asterisks (*, #) represent p < 0.05 as compared to vehicle treated and STZ-HG/STZ-NG rats respectively.

We hypothesize that STZ has a direct effect on TRPV1 expression and function in the central terminals of the spinal cord. STZ-induced increase in microglial activation and pro-inflammatory mediator levels can alter the expression levels or sensitize TRPV1, which can increase the release of CGRP from the presynaptic sensory nerve terminals. In the next set of experiments, we used RTX, a potent TRPV1 agonist, when given intrathecally, can specifically ablate TRPV1 expressing central terminals of sensory neurons. In order to understand the role of spinal TRPV1 in STZ-induced hyperalgesia, we administered RTX (2 μg/kg/20 μl) intrathecally 2 weeks after STZ treatment. RTX significantly prevented thermal hyperalgesia in both STZ-HG (Week 5: vehicle,11.3 ± 0.5 s; STZ-HG,8.0 ± 0.3 s; STZ-HG (RTX-treated), 11.0 ± 0.2 s, n = 12, p > 0.05) and STZ-NG animals (Week 5: vehicle,11.3 ± 0.5 s; STZ-NG,9.2 ± 0.3 s, STZ-NG (RTX-treated),12.5 ± 0.60 s, n = 12, p > 0.05) (Figure 7A, B). Intrathecal RTX did not cause any change in mechanical hypersensitivity in both STZ-HG (Week 5: vehicle, 36.8 ± 1.6 g; STZ-HG,24.4 ± 2.0 g; STZ-HG (RTX-treated),25.0 ± 2.3 g, n = 8, p < 0.05) and STZ-NG animals (Week 5: vehicle,36.8 ± 1.6 g; STZ-HG,23.0 ± 2.6 g; STZ-HG (RTX-treated), 26.4 ± 1.1 g, n = 8, p < 0.05) (Figure 7C, D), suggesting the involvement of TRPV1 only in thermal hyperalgesia. This observation is consistent with the findings that TRPV1 selectively mediates inflammatory thermal hypersensitivity, but not mechanical hypersensitivity.

## Discussion

In the present study, we have characterized the metabolic state, release of inflammatory mediators, and TRPV1 expression and function in the spinal cord following the administration of STZ. The major finding of the study is that STZ-induced early phase of thermal hyperalgesia (up to 5 weeks) is independent of glycemic state and the ability of pancreatic β-cells to release insulin, but is correlated with microglial activation (increased OX-42 staining), increased TRPV1 expression, and increased levels of inflammatory mediators (TNF-α, IL-6, and IL-1β) in spinal cord. In both STZ-NG and STZ-HG rats, TRPV1 expression in spinal dorsal horn was significantly increased as shown by TRPV1 immunostaining and TRPV1-mediated CGRP-release. Further evidence for the involvement of TRPV1 in early phase of hyperalgesia was deciphered by the reversal of thermal hyperalgesia, but not mechanical hyperalgesia after intrathecal administration of RTX, a potent TRPV1 agonist that is known to ablate TRPV1 expressing nerve terminals in the spinal cord [27,29-31,35].

Studies from other laboratories and ours have shown differential sensitivity to administration of the same dose of STZ within the same strain of animals [12,36,37]. The reason for this difference is far from clear. This could be due to differences in the expression of β-cell-specific Glut-2 isoform of glucose transporter (responsible for the β-cell-specific uptake of STZ) [37]. Final stages of STZ-induced cell death cascade involve activation of islet cell poly (ADP-ribose) polymerase, the expression levels of which may differ within a strain [38]. Studies have also shown that time lapsed between the last meal and STZ injection and the size of the pancreas could contribute to the variation. Other factors that may be responsible include: insufficient concentration of STZ reaching the pancreas, faster recovery of damaged pancreatic β-cells, and a decreased bioavailability of STZ as a result of rapid metabolism and excretion. In this study, we have observed a similar incidence of developing hyperglycemia (60% hyperglycemic) following STZ administration as seen with wild-type and age-matched single transgenic mice (Ins-HA.D2 mice) [12]. The incidence of hyperglycemia could be correlated with the changes in the body weight, plasma insulin levels and glucose tolerance in these animals. Vehicle-treated and STZ-NG rats showed a normal gain in the body weight, whereas there was a significant decrease in the body weight of STZ-HG rats, suggesting that STZ-NG rats do not suffer from metabolic derangement. There was no change in plasma insulin levels in STZ-NG as compared to vehicle treated rats. It is interesting to note that the blood glucose levels were slightly higher following glucose tolerance test in STZ-NG animals. This suggests a compromised β-cell function in STZ-NG as compared to vehicle-treated rats.

The present study revealed that the time course and the degree of thermal and mechanical hypersensitivity were similar in both STZ-HG and STZ-NG rats, regardless of their glycemic state. Thermal hyperalgesia was evident within a week, whereas mechanical allodynia was seen 3 weeks after STZ treatment. Interestingly, the profile was similar in both STZ-HG and STZ-NG rats. This has not been documented in the literature, may be because of the common practice of excluding STZ-NG animals from the studies or injecting a booster dose of STZ. This observation raises a puzzling question whether change in thermal and mechanical sensitivities observed as a consequence of hyperglycemia or a direct effect of STZ. Previously, we have reported this phenomenon in mice and suggested that thermal hypersensitivity observed in a mouse model could result from the direct neuronal effects of STZ leading to enhanced expression and function of TRPV1 receptor in DRG [12]. There are evidences in the literature that hyperglycemia and hyperalgesia are not correlated [39]. Studies have shown that < 40 to 60% of rats injected with STZ were not hyperglycemic but exhibited mechanical hyperalgesia [13,40,41]. Diabetes associated disturbance of autonomic function and pain sensation in STZ-treated animals were independent of glycemic factors or dyslipidemia [13]. Studies have shown a lack of correlation between acute hyperglycemia and thermal hypersensitivity, warmth/cooling sensation, pressure pain, von Frey filament thresholds or vibration perception thresholds [40,42-44]. Furthermore, pain pressure thresholds in type 2 diabetic Zucker rats could be corrected with insulin-like-growth factor II (IGF-II) that has no effect on blood glucose levels [45]. Based on our findings in mice, we have proposed that a direct action of STZ plays a role [12]. Therefore, we decided to focus on the possible mechanisms underlying hyperalgesia.

STZ-treated rats exhibited microglial activation, indicated by enhanced OX-42 staining and enhanced release of pro-inflammatory cytokines in spinal cord. This was independent of the glycemic state. It has been suggested that STZ-treated animals show microglial activation [16,18]. In this study, we report for the first time that STZ-NG animals show microglial activation. Further, levels of IL-1β, IL-6, and TNF-α in spinal cord tissue were significantly higher in STZ-HG and STZ-NG animals. Microglial activation in spinal cord may cause pro-inflammatory cytokine release. Several lines of evidence suggest that cytokines have pro-nociceptive actions, which are mediated by the sensitization of TRP channels. The production and release of IL-6 by retinal microglia as well as corneal and retinal epithelial cells is partially dependent on the activation of TRPV1 [46-48]. IL-1β can induce pain hypersensitivity by activating TRPV1-positive nociceptors via a p38-MAPK dependent mechanism [49]. TNF-α may have a significant impact on nociceptive signaling at the spinal cord level, mediated by increased responsiveness of presynaptic TRPV1 receptors to endogenous agonists [23]. Long-term exposure of primary afferent neurons to TNF-α significantly increased the proportion of TRPV1-immunoreactive neurons [50]. TRPV1 activation increases TNF-α receptor 1 expression in cultured mouse DRG neurons through a ROS signaling pathway [51,52]. Further, C-C motif chemokine-2 (CCL2) is co-localized with TRPV1 in the spinal dorsal horn [53,54]. Intrathecal administration of CCL2 produced thermal hyperalgesia, which was inhibited by selective CCL2 receptor antagonist (INCB3344) and a specific CCL2 neutralizing antibody [54,55]. Upregulation of CCL2 receptor may contribute to TRPV1 expressing neuronal excitability [56]. TRPV1 may also be involved in releasing inflammatory agents from immune cells [15]. These studies provide evidence that increased levels of pro-inflammatory cytokines/chemokines can sensitize TRPV1 and at the same time over expression of TRPV1 can augment the release of these cytokines/chemokines. TRPV1 expression has been shown in microglia and astrocytes [57]. TRPV1 activation on the microglia/neuronal terminals can lead to influx of Ca^2+ ^and cause glutamate and CGRP release, which can subsequently activate glia [27,28,32,58,59]. The interplay between microglial activation, increased expression/function of TRPV1 and neurotransmitter/neuromodulator release may contribute to central sensitization resulting in the development of hyperalgesia.

Finally, we investigated whether TRPV1 expressed in the central terminals of sensory neurons in the spinal cord is involved in mediating thermal and mechanical hypersensitivities seen in STZ-HG and STZ-NG animals. In order to achieve this, we chose a specific TRPV1 agonist RTX, which can selectively impair TRPV1, functions in the spinal cord. Intrathecal RTX administration attenuated the development of STZ-induced thermal hyperalgesia, but had no effect on mechanical hypersensitivity. TRPV1 has been shown to be involved only in inflammatory thermal hypersensitivity, but not in mechanical hypersensitivity [27,29,35,60,61]. Analgesic effects of localized application of RTX can be explained by its ability to cause depolarization block of the peripheral or central nerve terminals in the short-term and nerve terminal ablation in the long-term [26,27,32]. These observations further validate the claim that TRPV1 is involved in inflammatory thermal but not mechanical hypersensitivity.

## Conclusions

In conclusion, we have shown in this study that STZ, independent of its ability to cause β-cell damage and hyperglycemia, can cause activation of microglia, and release pro-inflammatory agents in spinal dorsal horn. This further enhanced the expression of TRPV1 and influx of Ca^2+^, leading to the development of central sensitization and contributes to thermal hyperalgesia. Attenuation of thermal hyperalgesia and TRPV1-mediated CGRP release by intrathecal administration of RTX confirm the role of TRPV1 in STZ-induced inflammatory thermal hyperalgesia.

## Methods

### STZ-administration

All procedures used in this study were approved by the animal care and use committee at Southern Illinois University, School of Medicine, and conformed according to National Institutes of Health and institutional guidelines. Experiments were conducted on male Sprague Dawley (275-300 g) rats purchased from Harlan laboratories (Indianapolis, IN, USA). Rats were housed in specific pathogen-free barrier animal facility, and rodent laboratory chow (Laboratory Diet 5001; Nutrition International, Inc., Brentwood, MO) and drinking water were provided ad libitum. Rat housing was maintained on a 12-h light/dark cycle at an ambient temperature of 22 ± 1°C. Freshly prepared STZ (55 mg/kg) in saline (pH 4.5 with 0.1 N citrate buffer) was injected intraperitoneally as described previously [12,62]. Vehicle rats (n = 10) received citrate buffered saline alone. In first set of experiments, 20 animals were injected with STZ. Two days after STZ injection, animals were divided in three different groups on the basis of blood glucose levels; vehicle (control animals treated with saline), STZ-hyperglycemic (STZ-HG) (blood glucose level > 300 mg/dl) and STZ-normoglycemic (STZ-NG) (blood glucose level < 120 mg/dl). After 5 week of blood glucose level measurements and behavioural pain testing (per week) the animals were sacrificed to carry out *in-vitro *studies. In second set of experiments, 20 animals were injected STZ to study the effect of RTX on STZ-induced hyperalgesia. Glucose levels were determined with an OneTouch Ultra blood glucose monitoring system (LifeScan, Milpitas, CA) using whole blood obtained from the tail.

### RTX-administration

The experimental procedures especially in reference to animals not experiencing unnecessary discomfort, distress, pain or injury have been approved by the Southern Illinois University School of Medicine Institutional Animal Care and Use committee review panel in accordance with the Panel of Euthanasia of American Veterinary Medical Association. Two days after STZ injection animals were divided in three different groups as described previously. Animals were tested for thermal and mechanical sensitivities for 2 weeks (once/week). After 2 weeks, rats in STZ-HG and STZ-NG groups received intrathecal (L4/L5 intraspinal space) injection of 2 μg/kg/20 μl of RTX in saline under isoflurane anesthesia (2% oxygen) [29,63]. Control group received a corresponding volume of vehicle. All the rats were acclimatized to the test conditions 1 hour/day for 5 days before starting the experiments.

### Behavioural assays

#### Measurement of thermal sensitivity

Thermal nociceptive responses were determined using a plantar test instrument (Ugo Basile, Camerio, Italy). The rats were habituated (15 minutes acclimation period) to the apparatus that consisted of three individual Perspex boxes on a glass table. A mobile radiant heat source was located under the table and focused onto the desired paw. Paw withdrawal latencies (PWLs) were recorded three times for each hind paw and the average was taken as the baseline value. A timer was automatically activated with the light source, and response latency was defined as the time required for an abrupt withdrawal of the paw. The apparatus has been calibrated to give a normal PWL of approximately 10-12 s. In order to prevent tissue damage, an automatic cut-off at 20 s was set.

#### Measurement of mechanical sensitivity

Mechanical nociceptive responses were assessed using a dynamic plantar anesthesiometer instrument using von Frey hairs (Ugo Basile, Camerio, Italy). Each rat was placed in a chamber with a metal mesh floor and was habituated (15 minutes acclimation period) to the apparatus. A 0.5 mm diameter von Frey probe was applied to the plantar surface of the rat hind paw with pressure increasing by 0.5 g/s and the pressure at which a paw withdrawal occurred was recorded and this was taken as Paw Withdrawal Threshold (PWT). For each hind paw, the procedure was repeated 3 times and the average pressure to produce withdrawal was calculated. Successive stimuli were applied to alternating paws at 5 min intervals. All the experiments involving behavioral studies were performed by the experimenter who was unaware of treatments.

### Immunostaining

Five weeks after STZ injections, immunofluorescence labeling of TRPV1 receptors and OX-42 (microglial marker) in spinal dorsal horn was performed on three vehicle-treated, three STZ-HG and three STZ-NG rats to determine the effect of STZ administration on TRPV1 receptor expression and microglial activation in spinal dorsal horns. Rats were deeply anesthetized with i.p. injection of ketamine (85 mg/kg) and xylazine (5 mg/kg) and perfused intracardially with 100 ml ice-cold normal saline followed by 150 ml 4% paraformaldehyde in 0.01 M PBS (pH 7.4). The lumbar segment of the spinal cord were quickly removed and post-fixed for 2 hr in the same fixative solution and cryoprotected in 20% sucrose in 0.01 M PBS for 24 hr at 4°C. Following cryoprotection, 15 μm sections were cut from lumbar portion of spinal cords using a microtome (Leica CM 1850, North Central Instruments Inc, Plymouth MN, USA).

For TRPV1 receptor and microglial marker (OX-42) immunostaining, the sections were rinsed in 0.1 M PBS and permeated with 1% triton X in 0.01 M PBS for 30 minutes. The sections were then blocked in 10% normal donkey serum in PBS for another 30 minutes. The sections were then incubated overnight with the primary antibody (TRPV1: Guinea anti-TRPV1 N terminal (dilution 1:1000) Neuromics, Minneapolis, MN, USA and microglial activation marker: Mouse anti-CD11b (OX-42) (dilution 1:100), Novus Biologicals, Littleton, CO, USA) diluted in PBS containing 1% normal donkey serum, 0.1% Triton X-100. Subsequently, sections were rinsed in PBS and incubated (1 hour) with the secondary antibody (Rhodamine donkey anti guinea pig IgG (dilution 1:50), Jackson Immunoresearch Laboratories, PA, USA) diluted in PBS containing 1% normal donkey serum, 0.1% Triton X-100. Sections were washed and fixed. A confocal microscope (Specifications: Intensity-20%, Gain-1(4 for microglia slides), Zoom-1, Offset-0, Scan speed-150-160 seconds, Filter and color tool-normal) was used to view the sections, and areas of interest were photo documented and quantified. The results were expressed as average gray value/10,000 μm square area. Three to five sections from 3 different animals were analyzed.

### Neuropeptide release assays

#### Tissue preparation

Five weeks after STZ injections, five rats from each group i.e. vehicle-treated, STZ-HG, STZ-NG, STZ-HG RTX treated, STZ-NG RTX treated were anesthetized using isoflurane (5%) and oxygen. Spinal cord was collected quickly by hydraulic method. Lumbar portion of spinal cord were collected and washed for 30 min in synthetic interstitial fluid (SIF) (107.8 mM NaCl, 26.2 mM NaCO_3_, 9.64 mM Na-gluconate, 7.6 mM sucrose, 5.05 mM glucose, 3.48 mM KCl, 1.67 mM NaH_2_PO_4_, 1.53 mM CaCl_2_, 0.69 MgSO_4_) gassed with 95% oxygen and 5% carbon dioxide at 32°C. Three segments each of 0.10 g (range 0.05-0.15 g) from lumbar spinal cord of each animal were taken for further elution studies (Bishnoi et al., 2011).

#### Elution procedures and stimulation

A series of 3 glass tubes were filled with 0.5 ml SIF each and positioned in a temperature controlled (32°C) shaking bath. The release experiment was started by transferring the mounted spinal cord segments into the first tube. After 5 min incubation, tissues were transferred to the second tube for another 5 min and then moved to the third tube containing capsaicin (10^-5 ^M, (8-methyl-*N*-vanillyl-6-nonenamide, Sigma Aldrich, St Louis, MO, USA) for 5 minutes. Capsaicin was prepared as stock solutions in ethanol (100 mM for capsaicin) and diluted to desired concentrations using physiological solution prior to the experiments. The control experiments were performed with the equivalent amounts of the solvent. All the solutions were freshly prepared before the experiments.

#### Enzyme immunoassays

For CGRP release assay, 100 μl of elutes were taken from each tube. Spinal cord elutes were further centrifuged at 2000 rpm for 10 minutes. Samples were mixed immediately after the incubation with 400 μl (1:4 dilution) of commercial CGRP EIA buffer containing several protease inhibitors. The CGRP content was determined immediately after the end of the experiment using commercially available enzyme immunoassays (CGRP: Bertin Pharma (formerly SPlbio), France, limit of detection = 0.7 pg/ml, range = upto 500 pg/ml). All EIA plates were determined photometrically using a microplate reader.

### Measurement of pro-inflammatory mediators in spinal cord homogenates

Five weeks after STZ injections, five rats from each group (vehicle treated, STZ-HG and STZ-NG) were sacrificed. Spinal cord from each animal was collected quickly by hydraulic method. Lumbar portion of spinal cord were collected and cut into pieces. Tissues were homogenized in cell-lysis buffer using hand homogenizer and centrifuged. The supernatant was collected to measure the levels of pro-inflammatory mediators (IL-1β, IL-6, TNF-α) using respective ELISA kits (IL-1β, IL-6, TNF-α: Rat IL-1β ELISA kit, Rat IL-6 ELISA kit and Rat TNF-α ELISA kit were purchased from Invitrogen Corporation, Camarillo, CA, USA) following manufacturer instructions.

### Statistical analysis

Statistical analysis was performed using SPSS software. Data are presented as mean ± SEM. Descriptive one way analysis of variance (ANOVA) was used to compare PWL and PWT at given time point for vehicle and/or STZ and/or RTX treated animals. Descriptive one way ANOVA followed by post hoc Tukey's HSD was used to compare more than 2 groups. A p value of < 0.05 was considered as statistically significant.

## Abbreviations

DPN: Diabetic Peripheral Neuropathy; DRG: Dorsal Root Ganglion; EPSC: Excitatory Post Synaptic Currents; ERK: Extracellular Signal-regulated Protein Kinase; IL-6: Interleukin6; IL-1β: Interleukin1β; MAPK: Mitogen Activated Protein Kinase; ROS: Reactive Oxygen Species; TNF-α: Tumor Necrosis Factor α.

## Conflict of interests

The authors declare that they have no competing interests.

## Authors' contributions

MB performed the drug administration and immunofluorescence and drafted the manuscript. CB performed the behavioral tests. MA and LZ performed the ELISA. LP designed the study and drafted the manuscript. All authors read and approved the final manuscript.
